# Training With Virtual Patients in Transcultural Psychiatry: Do the Learners Actually Learn?

**DOI:** 10.2196/jmir.3497

**Published:** 2015-02-16

**Authors:** Ioannis Pantziaras, Uno Fors, Solvig Ekblad

**Affiliations:** ^1^Cultural Medicine UnitDepartment of Learning, Informatics, Management and Ethics (LIME)StockholmSweden; ^2^Department of Computer and Systems SciencesStockholm UniversityStockholmSweden

**Keywords:** mental health, transcultural psychiatry, virtual systems, PTSD, medical informatics, education, patient simulation

## Abstract

**Background:**

The rapid increase in the number of patients with diverse ethnic backgrounds and previous exposure to severe mental trauma dictates the need for improvement in the quality of transcultural psychiatric health care through the development of relevant and effective training tools.

**Objective:**

This study aimed to evaluate the impact of training with a virtual patient on the learner’s knowledge of posttraumatic stress disorder symptoms, clinical management, and basic communication skills.

**Methods:**

The authors constructed an interactive educational tool based on virtual patient methodology that portrayed a refugee with severe symptoms of posttraumatic stress disorder and depression. A total of 32 resident psychiatrists tested the tool and completed a pre-interaction and post-interaction knowledge test, including skills, at the time and several weeks later.

**Results:**

All of the participants (N=32) completed the pre-interaction and post-interaction test, and 26 (81%) of them completed the online follow-up test. The mean pre-interaction score was 7.44 (male: 7.08, female: 7.65, no statistical significance). The mean post-interaction score was 8.47, which was significantly higher (*P*<.001) than the pre-interaction score (mean score 7.44). The mean score for the follow-up test several weeks later was 8.38, higher than the pre-interaction score by 0.69 points but not statistically significant.

**Conclusions:**

Our results suggest that virtual patients can successfully facilitate the acquisition of core knowledge in the field of psychiatry, in addition to developing skills such as clinical reasoning, decision making, and history taking. Repeated training sessions with virtual patients are proposed in order to achieve sustainable educational effects.

## Introduction

The number of patients with diverse ethnic backgrounds (often having been exposed to severe mental trauma) is rapidly increasing [1,2], which highlights the crucial need for effective training platforms that can provide essential knowledge and skills to care for this vulnerable group. Immigrants have higher rates of disability than host populations: social disability including unemployment [3], mental disability such as posttraumatic stress disorder (PTSD) and major depression [4,5], as well as physical disability such as cardiovascular disease [6] and metabolic syndrome [7]. Unfortunately, trauma-related diagnoses are often missed in primary care [8]. The need for new methods of acquiring proper communication skills is considered crucial, since most current systems incorporate these important skills informally in clinical training under supervision but with no direct focus on them.

Rapid technological progress during the last few decades has enabled the development of innovative educational tools, most often to supplement traditional medical curricula. Virtual patients (VPs) are broadly defined as “interactive computer simulations of real-life clinical scenarios for the purpose of medical training, education, or assessment” [9]. They include a highly diverse group of platforms that, since the first published description in the early 1970s (10), have been developed and studied thoroughly and shown to provide a realistic, reliable, safe, and consistent learning environment for enhancing various aspects of knowledge and skills, including clinical reasoning, clinical decision making, communication, and history taking [10-15].

Very few studies have explored implementation of VPs by psychiatrists [16-19], and to the best of our knowledge, no other VP system dedicated to traumatized refugees has been described, although virtual reality has recently been used and studied as a means of providing exposure therapy for veterans with combat-related PTSD [20]. Previous papers published by our research team examined our system in terms of various aspects of user acceptance, expectations, attitudes, and educational potentials [21-23] and obtained promising results. This study examined the impact of a training session with the VP system on core knowledge related to PTSD symptomatology and clinical management, as well as basic communication skills.

## Methods

### Refugee Trauma Simulation System

**Figure 1:**
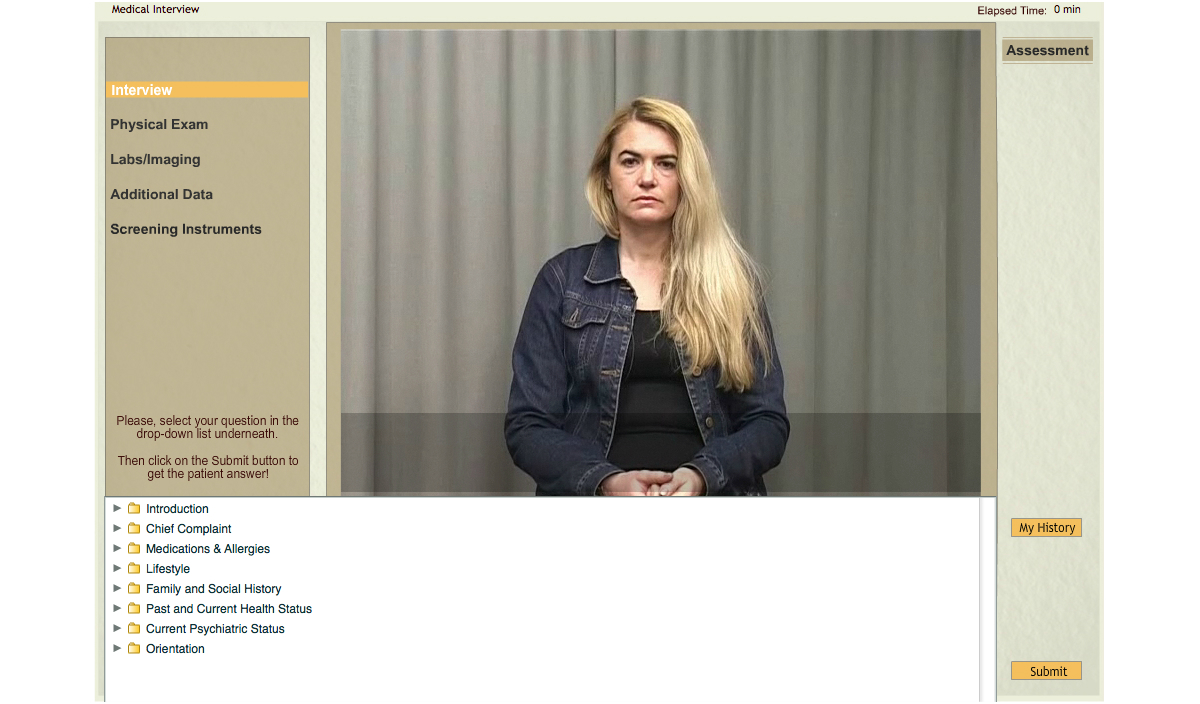
Screenshot of the RT-Sim system presenting the history-taking interview module.

**Figure 2:**
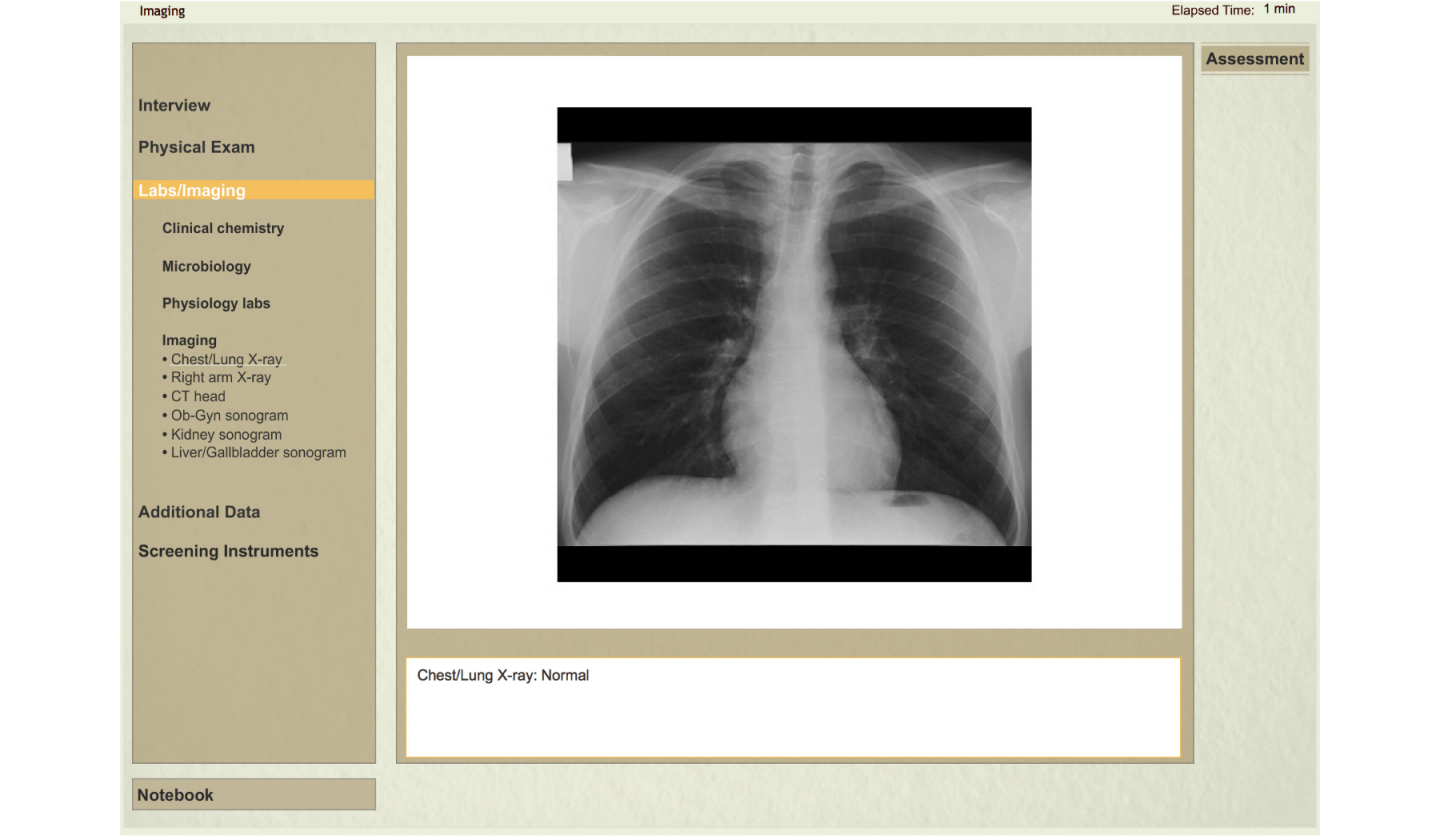
Screenshot of the RT-Sim system presenting the laboratory and imaging module.

We developed a VP system called Refugee Trauma Simulation (RT-SIM) that portrays an adult Bosnian refugee (“Mrs K.”) who presents severe symptoms of PTSD and major depression (Figure 1). The VP is shown in video format by displaying appropriate prerecorded sequences depending on the questions asked by the user. The medical interview is conducted by selecting questions from a list based on suitable categories. The user can physically examine the VP, as well as order laboratory and imaging tests (Figure 2). A preliminary assessment, including a summary of the patient’s history, differential diagnosis, and treatment plan is filled out by the learner upon completion of the virtual consultation.

An individualized, automated feedback module provided by both the VP and a virtual advisor (VA) follows. The feedback by the VP gives the patient’s perspective of the consultation, while the feedback by the VA focuses on more technical and clinical aspects of PTSD diagnostic criteria, clinical management, and basic communication skills. The feedback is designed in accordance with the learner’s actual performance and provides a brief and relevant theoretical background.

For example, if the learner did not sufficiently examine the patient’s trauma history, the VA commented: “I do not think that you sufficiently examined the patient’s trauma history by asking enough relevant questions about traumatic events. This is important in order to investigate exposure to events that could involve actual or threatened death or serious injury, or a threat to the physical integrity of self or others. If the patient reacted with intense fear, helplessness, or horror during such exposure, the first criterion for PTSD according to DSM-IV has been fulfilled”. A more detailed description of the construction of the feedback module was presented in a previously published study [23].

### Design and Evaluation Outcomes

In response to email invitations sent by the department heads at three major university hospitals in Sweden, 32 psychiatric residents agreed to participate in our study. The participants sat in silent group rooms under supervision and were asked to interact with the VP for up to 45 minutes as they would in a real-life clinical encounter.

The pre-interaction and post-interaction outcome was a knowledge test consisting of 11 multiple-choice questions that were linked to the following fields: diagnostic criteria of PTSD according to the *Diagnostic and Statistical Manual of Mental Disorders*, 4th edition (DSM-IV), clinical management of patients with PTSD, and theoretical aspects of basic communication skills. The highest possible score for each test was 11 (each correct answer generating 1 point). Multiple response items, for which more than one answer was keyed as correct, were so indicated. A follow-up test, hosted by the Karolinska Institutet online learning management system (Ping-Pong), was given several weeks later. All three tests (pre-interaction, post-interaction, and follow-up) consisted of identical multiple-choice questions, though appearing in different orders. After completion of the tests, no feedback about the success rate or correct answers was provided to the participants. Explicit instructions stated that no outside help was allowed during the follow-up online test. An advisory opinion by the Stockholm Regional Ethical Review Board (2011/321-31/3) was obtained for this study, and informed consent was obtained from all of the participants.

### Statistical Analysis

Stata/IC 12.1 for Mac was used for statistical analysis of the data. We used descriptive statistics to analyze demographic data and the matched-pair *t* test in order to estimate changes in the scores between pre-interaction, post-interaction, and follow-up tests. Participants who did not complete the follow-up test were excluded from that particular analysis. *P* values ≤.05 were regarded as evidence of statistical significance.

## Results

All of the participants (N=32; 12 males, 20 females) completed the pre-interaction and post-interaction knowledge test. Twenty-six (81%) of the participants completed the online follow-up knowledge test (9 males, 17 females). The mean age was 35.6 (female: 35.5, men: 35.9; range 28-51), while their mean experience as psychiatric residents was 2.3 years (female: 2.4, men: 2.1; range 0-5). No statistically significant differences in age and work experience between male and female participants were found. The mean duration of the interactive session with the patient was 68 minutes (min: 25; max: 110). The mean time between the interactive session with the VP and the online follow-up test was 79 days: 78 days for men and 79 days for women (not significant, *P*=.78).

**Table 1:** Scores on pre-interaction (Pre), post-interaction (Post), and follow-up (FU) knowledge test, overall, and by gender.

Participants	Data	N	Mean	SD	CI	*t*	df	*P*
**All**								
	Pre	32	7.44	0.31	6.8-8.07			
	Post	32	8.47	1.65	7.86-9.06			
	FU	26	8.38	2.02	7.57-9.20			
	Pre vs Post		-1.03	1.33	-1.51 to -0.55	-4.38	31	<.001^a^
	Post vs FU		0.35	1.74	-0.36 to 1.05	1.01	25	.32
	Pre vs FU		-0.69	2.29	-1.61 to 0.23	-1.54	25	.14
**Male**								
	Pre	12	7.08	2.02	5.80-8.37			
	Post	12	7.92	2.02	6.63-9.2			
	FU	9	8	2.45	6.12-9.89			
	Pre vs Post		-0.83	1.19	-1.59 to -0.08	-2.42	11	.03^a^
	Post vs FU		0.11	2.02	-1.45 to1.67	0.16	8	.87
	Pre vs FU		-0.67	2.00	-2.20 to 0.87	-1.00	8	.35
**Female**								
	Pre	20	7.65	1.60	6.90-8.40			
	Post	20	8.80	1.32	8.18-9.42			
	FU	17	8.59	1.80	7.66-9.52			
	Pre vs Post		-1.15	1.42	-1.82 to -0.48	-3.61	11	.002^a^
	Post vs FU		0.47	1.62	-0.36 to 1.31	1.19	8	.25
	Pre vs FU		-0.71	2.49	-1.99 to 0.58	-1.17	8	.26

^a^Statistically significant.

Table 1 shows the mean scores on the pre-interaction, post-interaction, and follow-up knowledge test, both overall and by gender. The mean pre-interaction score was 7.44 (male: 7.08, female: 7.65, non significant), and the mean post-interaction score was 8.47, demonstrating an improvement of 1.03 points, which was highly statistically significant overall (*P*<.001) and by gender (male: *P*=.03, female: *P*=.002). The mean score on the follow-up knowledge test was 8.38, higher than the pre-interaction score by 0.69 points, which was not statistically significant. The follow-up score was 0.35 points lower than the post-interaction score, which was not statistically significant. Similar results were found when the data were analyzed by gender.

## Discussion

### Principal Findings

This paper describes a VP system that was constructed as a platform for training clinical management of traumatized refugees and was evaluated in terms of impact on the learner’s core knowledge of PTSD symptomatology and clinical management, as well as basic communication skills.

To the best of our knowledge, only one published study has previously evaluated how VPs facilitate core knowledge in the field of psychiatry in general, finding no change in knowledge of PTSD symptoms pre-intervention and post-intervention [24]. Our results indicated a highly significant improvement on the knowledge test immediately after the training session with the VP. The fact that our participants did not receive any feedback about the success rate or correct answers after completion of the tests dramatically reduces the possibility that the improvement was due to recall rather than actual knowledge gain. The results were better on the follow-up test than the pre-interaction test but had declined and the improvement was no longer statistically significant. As far as our rather small sample allows, we can therefore conclude that a single session with the VP is not sufficient to produce a long-term impact on knowledge, which is consistent with previous findings that improvements in the performance of diagnostic tasks due to short-term training are not long-lasting [25]. We propose research about the impact of repeated sessions over a course of several months on the durability of acquired knowledge.

It has been suggested that VPs are not an ideal methodology for facilitating core knowledge, given potential cognitive overload and the fact that less interactive methods might be more effective [26]. Based on the system described above, we propose a VP model that combines experiential learning, as presented by Kolb [27], through active training in a realistic and appealing virtual environment, enhanced by short theoretical frameworks integrated into automated feedback. Our results support the hypothesis that this additional element can make VP systems valuable in the acquisition of core knowledge in addition to developing skills such as clinical reasoning and decision-making.

### Limitations

A limitation on the generalizability of our conclusions was the rather small sample of resident psychiatrists with similar work experience and expertise. Moreover, although the pre-interaction and post-interaction tests were conducted in a strictly controlled environment with no access to outside help, the possibility that participants, despite clear instructions, obtained such help during the follow-up test in an online, non-controlled environment cannot be ruled out.

For this prospective study, we used a single-subject design, which is common in applied fields of education and psychology for which subjects serve as their own controls [28]. This design can be effectively used to evaluate the impact of an intervention (such as virtual patients) that has not been widely studied and provide an initial clue as to its effectiveness before planning larger-scale studies with other designs and populations. However, a limitation of this methodology is its inability to know how a control group receiving another form of intervention would perform. On the other hand, a review [26] showed that it is difficult to make a comparison with a control group that receives traditional forms of education, such as lectures, in this area since clinical reasoning demands exposure to the actual situation (as provided by clinical practice or various forms of simulation).

### Conclusion

As a next step, we intend to conduct a randomized controlled trial that examines cognitive outcomes of using several VP cases, as well as actual patient outcomes. Future studies should also include the impact of working with the VP in pairs or groups, either at a local level or online.

## Abbreviations

DSM-IV: Diagnostic and Statistical Manual of Mental Disorders 4th edition
PTSD: posttraumatic stress disorder
VA: virtual advisor
VP: virtual patient
